# Correction: A tutorial on asymmetric electrocatalysis

**DOI:** 10.1039/d3cs90096g

**Published:** 2023-12-05

**Authors:** Jonas Rein, Samson B. Zacate, Kaining Mao, Song Lin

**Affiliations:** a Department of Chemistry and Chemical Biology, Cornell University Ithaca NY 14853 USA songlin@cornell.edu

## Abstract

Correction for ‘A tutorial on asymmetric electrocatalysis’ by Jonas Rein *et al.*, *Chem. Soc. Rev.*, 2023, https://doi.org/10.1039/D3CS00511A.

The authors regret that a cited author's name was spelled incorrectly in the main text of the original article. In the introductory paragraph of section 2 (A brief introduction to organic electrosynthesis) on page 8108, “Schotten and Williams” should be replaced with the correct spelling “Schotten and Willans”.

There are also two additional references which should have been included. On page 8113, the sentence which reads “Subsequent development of a catalytic system using K_3_Fe(CN)_6_ as a stoichiometric oxidant significantly enhanced the practicality of the system…” should read “Subsequent development of a catalytic system using *N*-methylmorpholine *N*-oxide^1^ or K_3_Fe(CN)_6_^2^ as a stoichiometric oxidant significantly enhanced the practicality of the system…”. These additional two references are shown below as ref. 1 and 2.

The Royal Society of Chemistry apologises for these errors and any consequent inconvenience to authors and readers.

## Supplementary Material
